# Dynamic Proteomics of Nucleus Accumbens in Response to Acute Psychological Stress in Environmentally Enriched and Isolated Rats

**DOI:** 10.1371/journal.pone.0073689

**Published:** 2013-09-09

**Authors:** Xiuzhen Fan, Dingge Li, Cheryl F. Lichti, Thomas A. Green

**Affiliations:** Center for Addiction Research, Department of Pharmacology and Toxicology, The University of Texas Medical Branch, Galveston, Texas, United States of America; Universidade de São Paulo, Brazil

## Abstract

Our prior research has shown that environmental enrichment (*i.e.* rats reared in an environment with novel objects, social contact with conspecifics) produces a protective antidepressant-like phenotype in rats and decreases neurobiological effects of acute psychological stress. Although CREB activity has been identified as a major player, the downstream molecular mechanisms remain largely unexplored. Thus, the current study investigates proteomic differences in the accumbens of rats raised in an enriched condition (EC) versus those raised in an isolated control condition (IC) under basal conditions and after 30 min of acute restraint stress. Results showed that under basal conditions, EC rats generally expressed less mitochondria-related proteins, particularly those involved in TCA cycle and electron transport compared to IC rats. After 30 min of acute stress, EC rats displayed **increased** expression of energy metabolism enzymes (among others) while IC rats exhibited **decreased** expression of similar proteins. Further, network and pathway analyses also identified links to AKT signaling proteins, 14-3-3 family proteins, heat-shock proteins, and ubiquitin-interacting proteins. The protein ENO1 showed marked differential expression and regulation; EC rats expressed higher levels under basal conditions that increased subsequent to stress, while the basal IC expression was lower and decreased further still after stress. The results of this study define differential protein expression in a protective rat model for major depression and additionally identify a dynamic and coordinated differential response to acute stress between the two groups. These results provide new avenues for exploration of the molecular determinants of depression and the response to acute stress.

## Introduction

Major Depression is one of the most widespread medical conditions in the United States. Lifetime prevalence rates are 16.5% (1 in 6 Americans; 50 million people) and last 12 month prevalence is about 6.7% (20 million Americans) [1]. Although pharmacotherapeutics have revolutionized the treatment of depression, many patients are refractory to pharmacotherapy [2]. Thus, a better understanding of the molecular mechanisms causing major depression is crucial for developing novel pharmacotherapeutic strategies to effectively treat this condition.

Although animal models cannot fully recapitulate the complex cognitive and social aspects of human depression, several models have proven useful both for developing pharmacotherapies and also furthering basic science into the mechanisms of depression [3]. Among these are the Forced Swim Test (FST) as a model of “behavioral despair”, the sucrose preference paradigm as a model of “anhedonia” and the social interaction test as a model of “social withdrawal”. These three tests are easy to perform in rodents and model the most common symptoms of depression.

The environmental enrichment paradigm is a non-drug and non-surgical manipulation of individual differences in rat behavior. Rats in the control condition, termed the Isolated Condition (IC) are singly housed in standard polycarbonate cages with no access to novelty or social contact. Rats in the Enriched Condition (EC) are housed 12 per group in a large cage with novel objects (hard plastic children’s toys). The EC rats experience social contact with conspecifics, exercise and novelty. The toys are changed daily to maximize novelty. After the toys are changed, the rats exhibit an incredibly high level of activity for at least 30 min as they explore the new toys, play and generally run around the cage. The activity they exhibit is much higher than locomotor stimulation seen with administration of any dose of cocaine or amphetamine (personal observation). After 30 min the activity levels wane and the rats eventually go back to sleep. However, as the light cycle transitions from light to dark, the rats show another robust burst of exploratory activity during their normal circadian activity peak. Interestingly, novelty, social contact and exercise are all rewarding to rats and all release dopamine in the mesolimbic pathway leading from the ventral tegmentum to the nucleus accumbens (NAcc) [4], [5], [6], [7], [8]. This “natural reward” pathway has received an incredible amount of attention in drug addiction studies [9], but has increasingly been studied for depression-related behavior [10]. **After a month in these differential rearing conditions EC and IC rats show robust differences in behavior. Specifically, EC rats exhibit a protective phenotype in depression-related behavioral paradigms, meaning that they behave as if they were injected with antidepressants** [11] **.** Thus, the innovative aspect of this study is the use of a rat model of a well described protective depression phenotype (*i.e.* EC/IC rats) to investigate the dynamic proteomics of differential responses to acute stress.

Our investigations into the molecular mechanisms underlying this protective phenotype has yielded evidence that EC rats have lower steady-state levels of the active phosphorylated form of the transcription factor cAMP response element binding protein (CREB) in the NAcc [11], [12]. The hypothesis that low CREB activity is the underlying mechanism of the EC phenotype has ample support by the fact that blocking CREB activity in the NAcc produces an EC-like phenotype in 14 different behavioral paradigms, including increased stimulant sensitivity, decreased stimulant self-administration, increased anxiety-like behavior and, of importance to this paper, decreased depression-like behavior [11],[13],[14],[15],[16],[17],[18]. Because of these prior reports, differentially-regulated CREB target genes are of highest importance in placing the current results in a proper context.

## Materials and Methods

### Materials

Precast immobilized DryStrips (pH 3–11NL, 11 cm) and IPG buffer (pH 3–11NL) were purchased from GE Healthcare (Uppsala, Sweden). 2D protein extraction buffer-III was purchased from GE Healthcare (Piscataway, NJ). SDS–Tris–HCI gradient gel (10–20%), coomassie blue G250 stain solution, protein solubilization buffer (PSB), were purchased from Bio-Rad (Hercules, CA). The other chemicals used were purchased from Sigma-Aldrich (St. Louis, MO) and were of analytical grade.

### Animal Treatment

Twenty four male Sprague Dawley rats (Harlan laboratories Inc, Houston), 21 days of age, were divided to two conditions (isolated control condition and enriched condition), twelve of each. For the IC group, the rats were separated one rat per cage with no access to social contact or novelty, whereas EC rats were housed together 12 per cage (77L×78W×60H cm) and with novel toys changed every day. The food and water were freely available for rats and all the rats were maintained in a controlled room environment (temperature, 22°C; relative humidity, 50%; and 12 h light/dark cycles) for 40 days (from post-natal days 21 to 61) prior to stress experimentation. The enrichment manipulation is a compound manipulation where rats experience social contact, exercise and novelty. All three of these aspects release dopamine in the nucleus accumbens and all are rewarding to rats [4], [5], [6], [7],[8]. Isolation rats were chosen as the control group rather than pair-housed rats because isolation is the absence of enrichment, and a pair-housed group would constitute an intermediately-enriched condition. Although it may appear that isolation produces a condition of chronic stress (as seen by elevated basal plasma corticosterone levels), enriched rats exhibit a blunted corticosterone response to stimulation. Chronic stress produces blunted corticosterone responses and increased sensitivity to stimulant-induced locomotor activity consistent with the idea that the life of an IC rat is *less* stressful than that of an EC rat [19], [20].

Restraint stress: Six rats from each group were placed individually into plastic conical sleeves (Decapi-Cone; model DC200; Braintree Scientific, Braintree, MA) for 30 min [11], [13], [14]. The other six rats in each group remained undisturbed as controls for stress. Stress rats were then decapitated immediately at the end of stress. The brain was chilled in cold phosphate-buffered saline (PBS) before being placed into a metal matrix (ASI Instruments) for dissection. A 2 mm thick coronal slice was taken using the olfactory tubercles as a guide. NAcc was dissected from the slice on an ice-cold platform using a razor blade. The olfactory tubercles and medial septal areas were removed and a triangular dissection of the NAcc was stored at −80°C until further analysis [21].

The experiments were performed in accordance with the guidelines of National Institutes of Health and approved by the Institutional Animal Care and Use Committee of The University of Texas Medical Branch at Galveston.

### Protein Extraction

The NAcc from two rats of the same group were pooled together in one tube for protein extraction due to its limited size. The tissue from controls and stress rats of both groups were first washed with ice cold tris buffered saline (TBS) and then homogenized in a buffer [TBS pH 7.4, 1% Igepal-CA630 (NP-40), 1×protease inhibitor cocktail, 20 mM NaF, 1 mM Na_3_VO_4_, 10 mM DTT and 5 mM ethylenediaminetetraacetic acid (EDTA)] on ice and then centrifuged at 750×g for 20 min at 4°C to remove the cell debris. The top fraction was transferred to a new tube and centrifuged at 20,000×g for 20 min at 4°C. The resulting supernatant was removed and mixed with 1% streptomycin sulfate to remove DNA contaminants [22], then, four volumes methanol and 1 volume of chloroform (V_protein_:V_methanol_:V_chloroform_ = 1∶4: 1) were added to the protein supernatant and incubated at room temperature 15 to 30 min., vortexing every 5 min to remove lipids. The sample was centrifuged at 16000×g, at 4°C for 20 min. The pellet was then washed with 3% HCI/acetone to remove the methanol and chloroform (cytosolic fraction) [23], [24], [25]; the procedure was similar for the resulting pellet fraction to obtain a crude membrane fraction. Both fractions were then dissolved in a buffer containing 6M urea, 1% NP-40, 20 mM,tris–HCl pH 7.4, 1×protease inhibitor cocktail, 10 mM dithiothreitol (DTT) and 5 mM EDTA. The protein extracts of both fractions were then subjected to further proteomic analysis.

### 2D Gel Electrophoresis

Four hundred micrograms of cytosolic protein from control and stress rats of both groups were dissolved in PSB 200 ul containing 100 mM DTT, a trace amount of bromophenol blue and 1% Immobilized pH Gradient (IPG) buffer, pH 3–11 NL, and incubated at 21°C for 1 hr. Three hundred fifty micrograms of protein from the membrane fraction were dissolved in 200 µl of 2D protein extraction buffer III containing 100 mM DTT, a trace amount of bromophenol blue and 1% IPG buffer, pH 3–11 NL, and incubated at 21°C for 1 hr. The proteins were then rehydrated to the DryStrip (11 cm, pH 3–11 NL) overnight at the same temperature. For the first dimension, isoelectric focusing (IEF) was performed at 20°C using an Ettan IPGphor3 (GE Healthcare, Sweden) in the following steps: 200 V for 30 min., 500 V for 1.5 hrs, 1000 V for 1.5 hrs, 8000 V for 2.0 hrs and 8000 V for 24000 Vhr. The strips were then equilibrated for 1 hr in equilibration buffer (50 mM Tris–HCl pH 8.8, 6 M urea, 20 mM iodoacetamide, 2% sodium dodecyl sulfate (SDS), 20% glycerol). After rinsing two times with SDS–polyacrylamide gel electrophoresis (PAGE) running buffer, the strips were loaded on to 10–20% SDS-Tris-glycine gradient gel (13.3×8.7×1×cm) and were then subjected to 150 V for 140 min at room temperature for the 2nd dimension separation [26]. Following the electrophoresis, the gel for each sample (N = 3 samples per group [i.e. 6 rats]) was stained with Coomassie blue G250 (CBB G250).

### SameSpots Analysis of Protein Expression Variation

2D gel images (CBB G250 stained) were acquired by Typhoon Trio imaging system (GE Healthcare). The scanning resolution was 50 µm with excitation λ 633 nm. The 2D gel images were then analyzed by the software Progenesis SameSpots (nonlinear dynamics, version 4.0) which has been judged to be much improved in reproducibility compared to previous generations of 2D gel analysis software [27]. To align the gel images, one of the six gels was chosen as a reference gel. With the help of manually drawn and automated vectors from each image to the reference, images were aligned at the pixel level. The program then performed automatic spot detection and background subtraction. The software assigns the same spot on every gel in the analysis with identical shape (spot outline) and spot number. Spot volumes were normalized to those of the reference gel to obtain normalized volumes that are comparable across gels. Protein expression fold changes between controls and stress rats in isolation and enrichment groups were determined as described below in the statistical analysis.

### Trypsin Digestion and MALDI-TOF-TOF MS Analysis

The regulated spots in both groups were manually excised from the 2D gel. The protein was digested with trypsin (0.1 µg per spot, Promega) in 10 µl of 25 mM ammonium bicarbonate, pH 8.0, for 6 hrs at 37°C [26]. One µl of digested sample solution was used for matrix-assisted laser desorption time of flight tandem mass spectrometry (MALDI TOF/TOF MS) analysis using an Applied Biosystems 4800 MALDI TOF/TOF proteomics analyzer. The instrument was operated in a positive ion reflection mode with mass range from 850 to 3000 Da. The focus mass was set at 1700 Da. For MS data, 2000–4000 laser shots were acquired and averaged from each sample spot. Following MALDI MS analysis, MALDI MS/MS was performed on several (∼5–10) abundant ions from each sample spot. A 1 kV positive ion MS/MS method was used to acquire data under post-source decay (PSD) conditions. The instrument precursor selection window was ±3 Da. For MS/MS data, 2000 laser shots were acquired and averaged from each sample spot. Automatic external calibration was performed using reference fragment masses 175.120, 480.257, 684.347, 1056.475, and 1441.635 (from precursor mass 1570.700).

### Protein Identification

Applied Biosystems GPS ExplorerTM (Version 3.6) software was employed for searching the respective protein database using both MS and MS/MS spectral data for protein identification. Protein match probabilities were determined by using MASCOT scores. A MASCOT score of more than 64 was considered significant (*i.e*. *P*<0.05). MS peak filtering included the following parameters: mass range 800 Da to 4000 Da, minimum S/N filter = 10, mass exclusion list tolerance = 0.5 Da, and mass exclusion list (for some trypsin and keratin-containing compounds) included masses 842.51, 870.45, 1045.56, 1179.60, 1277.71, 1475.79, and 2211.1. For MS/MS peak filtering, the minimum S/N filter = 10. The mass data were matched to the NCBI protein database. Precursor tolerance was set at 0.2 Da; MS/MS fragment tolerance was set at 0.3 Da; mass = monoisotopic; and peptide charges were only considered as+1.

### Statistical Analysis for Proteins

The intensities of same protein spots in 2D gels of basal expression and stress-evoked EC and IC protein changes were compared on log-transformed normalized volumes by Student’s *t*-test. *P* values <0.05 were considered significant. However, one spot was added at a *P* value of 0.06.

### Bioinformatics

First, basal and stress-evoked changes in proteins were analyzed *via* a gene/protein ontology procedure using the Database for Annotation, Visualization and Integrated Discovery (DAVID; http://david.abcc.ncifcrf.gov/) before being used in an *Ingenuity Pathways Analysis* (Ingenuity Systems; www.ingenuity.com).

#### Gene/protein ontology

Separate lists of proteins from Basal EC/IC Differences, IC Stress, and EC Stress were analyzed using the DAVID Functional Annotation Clustering tool. In this procedure, an algorithm clusters redundant and overlapping functional classifications to provide a clearer picture of protein functional regulation.

#### Ingenuity pathways analysis

The IPA suite was used to further analyze functional associations, canonical pathways and networks of interconnected proteins. Each of these analyses uses a Fisher’s exact test to identify overrepresentation of protein groups with a *P* value of less than 0.05. The Function analysis uses a similar method of classifying functions as the gene/protein ontology analysis, yet with a different set of comparison data. The Canonical Pathways feature searches for statistical overrepresentation of targets among curated pathways of direct and well-accepted biological systems (*e.g.* signaling cascades). The Network analysis feature is a more broad analysis of interactions among identified proteins mined from various literature sources.

### Western Blot Validation of Expression Changes

The same protein extracts used in 2D gel analyses (50 ug for ENO1 or 70 ug for HSPA8 and PKM2 per sample) were loaded in 10–20% SDS-Tris-glycine gradient gel (13.3×8.7 cm), and run at room temperature until the dye marker reached the end of the gel. The separated proteins were then transferred to PVDF membrane, and sequentially incubated with TBS with tween (TBST [pH 7.4, 0.1% Tween20 (sigma-Aldrich)]) with 5% nonfat milk for 1 hr; primary rabbit to rat specific antibody (ENO1, 1∶500; PKM2, 1∶1000; Cell signaling. HSPA8, 1∶1000; Epitomics) in TBST with 5% nonfat milk for 2 hrs; washing the membrane with TBST 15 min for three times; secondary dyelight550 conjugated goat to rabbit antibody (1∶15,000) in TBST with 5% nonfat milk for one hr; washing the membrane with TBST 15 min three times; then captured the image by Typhoon Trio (EX/EM, 532/580 nm, 50 um resolution) and analyzed by software ImageQuant TL 7.0. All these steps were performed at 21°C.

### Adenosine Triphosphate (ATP) Synthase Enzyme Activity

The ATP synthase activity and quantification were performed with an ATP synthase Specific Activity Microplate Assay Kit from ABCAM (ab109716). The STR tissue (with samples pooled as before) was homogenized in a buffer as described in the literature [28] to obtain the mitochondrial fraction. The F1F0 ATP synthase extraction from mitochondrial fraction was according to manufacturer’s instructions. Briefly, 10 ug per well of 10% detergent-extracts of protein was incubated in a 96-well plate at room temperature for 3 hrs to immuno-immobilize F1F0-ATP synthase, the enzyme hydrolyzing ATP to ADP and phosphate, and the ADP product is ultimately coupled to oxidized NADH to NAD^+^ which led to absorbance decreases at 340 nm. The reaction was monitored by Spectromax M2 microplate reader (Molecular Devices). Subsequently, ATP synthase protein (amount, not activity) was quantified using an antibody directed at ATP5B and coupled to alkaline phosphatase, which converts the pNPP substrate to a yellow color assayed at 405 nm.

### Possible Role for CREB in Protein Regulation

Because our prior research suggests EC/IC behavioral differences are due in large part to decreases in CREB activity in the NAcc of EC rats [11] and that blocking CREB activity produces an EC like behavioral phenotype in 14 different behavioral paradigms relating to addiction, depression, anxiety and natural reward [11], [12], [13], [14], [15], one might hypothesize many of the regulated proteins in this study would be CREB target genes. Accordingly, we utilized data from a published chromatin immunoprecipitation (ChIP) study [29] to identify which of our regulated proteins are CREB target genes. For our purposes, a CREB target gene is defined as P<0.005 in two or more ChIP experiments as listed in Table S4 of the Zhang et al. paper.

## Results

In this study, we investigated dynamic protein changes in the NAcc of EC and IC rats. We first analyzed differences in basal protein levels between EC and IC rats and then analyzed the rapid proteomic responses to restraint stress separately for EC and for IC rats. We employed gene/protein ontology analyses as well as Ingenuity Pathway Analyses to identify coordinated responses to stress.

### Basal Proteomic Differences between EC and IC Rats

**Figure 1:**
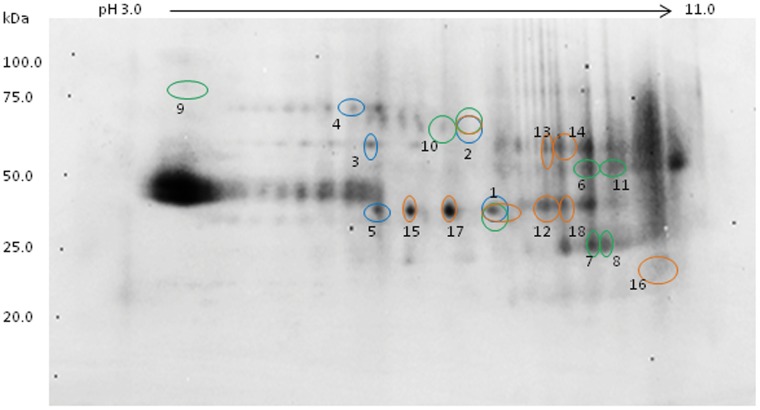
The expression of significantly regulated proteins from cytosolic extracts of rat NAcc (P<0.05) comparing EC vs. IC basal expression (blue), IC stress (green), and EC stress (orange), which were analyzed by Progenesis SameSpots in CBB stained gels.

**Figure 2:**
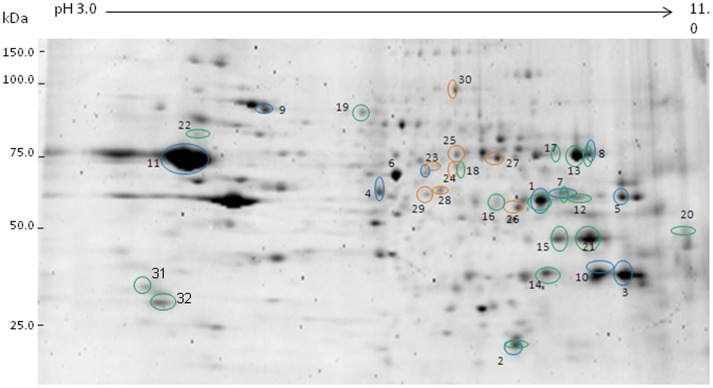
The expression of significantly regulated proteins from membrane extracts of rat NAcc (P<0.05) comparing EC vs. IC basal expression (blue), IC stress (green), and EC stress (orange), which were analyzed by Progenesis SameSpots in CBB stained gels.

Separate 2-DE gels were used for cytosolic and membrane fractions. The cytosolic fraction yielded 4 discrete proteins from 5 spots (see Figure 1; Table 1) and the membrane fraction yielded 10 separate proteins from 11 differentially expressed spots (see Figure 2; Table 2). It is notable that ENO1 was identified in two different spots with similar apparent molecular weight but significantly different pI. A similar situation occurred with VDAC1 except that the pI shift was not as dramatic.

**Table 1:** Proteins significantly regulated in the cytosolic fraction of rat NAcc in EC and IC rats.

Spot ID no.	Protein name	Gene symbol	NCBI No.	Number of matched peptides	Coverage (%)	MOWSE score	Fold change
***EC/IC Basal Differences***							
1	alpha-enolase isoform 1	ENO1	gi|158186649	8	29	258	1.2
2	dihydropyrimidinase-related protein 2	DPYSL2	gi|40254595	8	22	144	−1.9
3	chaperonin 60	HSPD1	gi|1778213	4	11	94	2.6
4	heat shock 70 kDa protein; Provisional	HSPA8	gi|149041392	4	24	129	3.2
5	Eno1 protein	ENO1	gi|38649320	6	19	133	2.2
***IC Stress***							
6	Chain A, Rat Liver F1-Atpase	ATP5A1	gi|6729934	10	23	578	−2.0
7	glyceraldehyde-3-phosphate dehydrogenase	GAPDH	gi|62653546	6	23	174	−1.8
8	glyceraldehyde-3-phosphate dehydrogenase	GAPDH	gi|62653546	4	20	207	−1.2
9	heat shock protein HSP 90-beta	HSP90AB1	gi|40556608	15	25	287	−1.8
10	dihydropyrimidinase-related protein 2	DPYSL2	gi|40254595	9	21	237	−2.1
2	dihydropyrimidinase-related protein 2	DPYSL2	gi|40254595	8	22	144	−1.4
11	ATP synthase, H+ transporting, mitochondrial F1 complex,alpha subunit, isoform 1	ATP5A1	gi|149029483	9	23	564	−1.6
1	alpha-enolase isoform 1	ENO1	gi|158186649	8	29	258	−1.2
***EC Stress***							
12	Alpha-enolase 1	ENO1	gi: 56757324	2	6	46	1.8
1	alpha-enolase isoform 1	ENO1	gi|158186649	8	29	258	1.4
13	pyruvate kinase isozymes M1/M2	PKM2	gi|16757994	9	24	295	1.8
14	pyruvate kinase isozymes M1/M2	PKM2	gi|16757994	12	37	372	2.0
15	enolase 3	ENO3	gi|109468300	8	27	262	1.7
16	ATP synthase subunit gamma, mitochondrial	ATP5C1	gi|39930503	4	21	189	2.4
17	Succinyl-CoA ligase [ADP-forming] subunit beta,mitochondrial	SUCLA2	gi: 52788305	8	15	264	1.5
18	Eno1 protein	ENO1	gi|38649320	7	24	84	1.6
2	dihydropyrimidinase-related protein 2	DPYSL2	gi|40254595	8	22	144	1.9

**Table 2:** Proteins significantly regulated in the membrane fraction of rat NAcc in EC and IC rats.

Spot ID no.	Protein name	Genesymbol	NCBI No.	Number of matched peptides	Coverage (%)	MOWSE score	Fold change
***EC/IC Basal Differences***							
1	creatine kinase U-type,mitochondrial	CKMT1A	gi|60678254	9	21	746	−1.6
2	Rieske Fe-S protein precursor	UQCRFS1	gi|206681	8	19	398	−4.1
3	voltage-dependent anion channel 1	VDAC1	gi|6755963	14	65	823	−1.7
4	NADH dehydrogenase [ubiquinone]iron-sulfur protein 2, mitochondrial.	NDUFS2	gi|58865384	18	50	441	−1.8
5	cytochrome b-c1 complex subunit 2, mitochondrial	UQCRC2	gi|55741544	14	39	512	−1.8
6	cytochrome b-c1 complex subunit 2, mitochondrial	UQCRC2	gi|55741544	11	32	192	−1.5
7	rCG42519, isoform CRA_a (citrate synthase)	CS	gi|149029697	8	19	427	−1.9
8	ATP synthase, H+ transporting mitochondrialF1 complex, alpha 1	ATP5A1	gi|149029483	21	54	948	−2.4
9	ATPase, H+ transporting, lysosomal V1subunit A	ATP6V1A	gi|157819953	29	51	675	−1.3
10	voltage-dependent anion channel 1	VDAC1	gi|6755963	14	65	479	−2.2
11	tubulin, alpha 1	TUBA1A	gi|6755901	14	42	591	1.5
***IC Stress***							
12	phosphoglycerate kinase 1	PGK1	gi|40254752	17	47	822	−2.1
7	citrate synthase	CS	gi|149029697	8	19	427	−2.0
13	ATP synthase, H+ transporting,mitochondrial F1complex	ATP5A1	gi|149029483	22	53	1,160	−1.7
14	voltage-dependent anion channel 1	VDAC1	gi|6755963	12	57	702	−2.0
15	glyceraldehyde-3-phosphate dehydrogenase	GAPDH	gi|8393418	11	43	411	−2.2
16	pyruvate dehydrogenase E1 alpha 1	PDHA1	gi|57657	9	21	258	−1.5
1	creatine kinase U-type,mitochondrial	CKMT1A	gi|60678254	14	33	746	−2.9
17	ATP synthase, H+ transporting mitochondrialF1 complex, alpha 1	ATP5A1	gi|149029483	16	44	408	−1.9
18	14-3-3 protein, gamma	YWHAG	gi|9507245	5	19	134	−1.8
2	Rieske Fe-S protein precursor	UQCRFS1	gi|206681	8	19	398	−3.2
19	Serum albumin, Precursor	ALB	gi|124028612	12	20	482	−1.2
20	NADH dehydrogenase [ubiquinone] 1alpha 9, mitochondrial.	NDUFA1	gi|81882598	15	43	445	−1.8
8	ATP synthase, H+ transporting mitochondrialF1 complex, alpha 1	ATP5A1	gi|149029483	21	54	948	−2.2
21	glyceraldehyde-3-phosphate dehydrogenase	GAPDH	gi|8393418	11	43	423	−1.9
22	rab GDI alpha	GDI1	gi|396431	13	37	147	2.4
31	tyrosine 3-monooxygenase/tryptophan5-monooxygenase activation protein,epsilon polypeptide, isoform CRA_a	YWHAE	gi|149053421	8	44	108	1.9
32	tyrosine 3-monooxygenase/tryptophan5-monooxygenase activation protein,zeta polypeptide	YWHAZ	gi|6756041	14	56	323	1.5 (P = 0.06)
***EC Stress***							
23	Unidentified						
24	Septin-11 protein	SEPT11	gi|38328220	8	14	266	1.7
25	sorting and assembly machinerycomponent 50	SAMM50	gi|51948454	9	21	198	1.7
26	Glutamate oxaloacetate transaminase 1	GOT1	gi|122065118	14	34	553	1.7
27	glutamate dehydrogenase 1, mitochondrialprecursor	GLUD1	gi|6980956	8	12	166	1.9
28	elongation factor Tu, mitochondrialprecursor	TUFM	gi|157820845	11	27	416	2.0
29	ARP1 actin-related protein 1 homolog A,centractin α	ACTR1A	gi|5031569	7	22	242	2.0
30	N-ethylmaleimide sensitive fusion protein,isoformCRA_b	NSF	gi|149054471	22	28	354	1.4

When proteins of the cytosolic and membrane fractions were added together, the gene/protein ontology analysis produced 5 main clusters of related features, specifically relating to mitochondrial membrane localization, oxidative phosphorylation, mitochondrial electron transport, nucleotide binding and mitochondrial matrix (see Table 3). The effect was such that the EC group had less expression of the proteins comprising the categories above. The overwhelming over-representation of mitochondria-related proteins is a theme repeated in the IC stress analysis below.

**Table 3:** Gene ontology terms for regulated proteins.

Category	Term	Count	%	*P* Value	Genes	Fold enrich-ment	Bonferroni	Benjamini	FDR
***EC/IC Basal Differences***									
**Annotation Cluster 1**		**Enrichment Score: 5.63**							
GOTERM_CC_FAT GO: 0005743	Mitochon-drial innermembrane	7	53.84	1.47E-07	UQCRC2, CKMT1A, ATP5A1, HSPD1, UQCRFS1, NDUFS2, VDAC1	22.49	1.25E-05	6.24E-06	1.58E-04
GOTERM_CC_FAT GO: 0005740	mitochondrial envelope	7	53.84	9.36E-07	UQCRC2, CKMT1A, ATP5A1, HSPD1, UQCRFS1, NDUFS2, VDAC1	16.42	7.95E-05	1.33E-05	0.001
**Annotation Cluster 2**		**Enrichment Score: 2.17**							
GOTERM_BP_FAT GO: 0006119	oxidative phosphorylation	4	30.77	5.83E-05	UQCRC2, ATP6V1A, ATP5A1, NDUFS2	46.01	0.016	0.0082	0.077
**Annotation Cluster 3**		**Enrichment Score: 2.03**							
SP_PIR_KEYWORDS	respiratory chain	3	23.08	8.66E-04	UQCRC2, UQCRFS1, NDUFS2	62.52	0.062	0.015	0.90
GOTERM_CC_FAT GO: 0005746	mitochondrial respiratorychain	3	23.08	0.0015	UQCRC2, UQCRFS1, NDUFS2	46.09	0.12	0.011	1.68
**Annotation Cluster 4**		**Enrichment Score: 1.95**							
SP_PIR_KEYWORDS	nucleotide-binding	6	46.15	0.0024	ATP6V1A, CKMT1A, ATP5A1, HSPD1, TUBA1A, HSPA8	5.27	0.16	0.025	2.49
**Annotation Cluster 5**		**Enrichment Score: 1.56**							
GOTERM_CC_FAT GO: 0005759	mitochondrial matrix	4	30.77	0.001	CS, ATP5A1, HSPD1, VDAC1	17.33	0.087	0.0083	1.15
GOTERM_CC_FAT GO: 0031980	mitochondrial lumen	4	30.77	0.001	CS, ATP5A1, HSPD1, VDAC1	17.33	0.087	0.0083	1.15
*IC stress*									
**Annotation Cluster 1**		**Enrichment Score: 3.63**							
GOTERM_BP_FAT GO: 0006091	generation of precursor metabolites and energy	8	47.06	1.82E-08	NDUFA9, CS, PDHA1, ATP5A1, PGK1, UQCRFS1, GAPDH, ENO1	21.61	5.52E-06	5.52E-06	2.44E-05
SP_PIR_KEYWORDS	glycolysis	4	23.53	6.13E-06	PDHA1, PGK1, GAPDH, ENO1	102.86	6.06E-04	3.03E-04	0.006785
**Annotation Cluster 2**		**Enrichment Score: 2.71**							
GOTERM_BP_FAT GO: 0006091	generation of precursor metabolites and energy	8	47.06	1.82E-08	NDUFA9, CS, PDHA1, ATP5A1, PGK1, UQCRFS1, GAPDH, ENO1	21.61	5.52E-06	5.52E-06	2.44E-05
GOTERM_CC_FAT GO: 0005739	mitochondrion	10	58.82	7.04E-07	YWHAZ, CKMT1A, NDUFA9, CS, PDHA1, ATP5A1, DPYSL2, UQCRFS1, YWHAE, VDAC1	7.35	5.63E-05	5.63E-05	7.48E-04
**Annotation Cluster 3**		**Enrichment Score: 2.34**							
SMART SM00101:	14_3_3	3	17.65	3.06E-06	YWHAZ, YWHAG, YWHAE	778.2	9.17E-06	9.17E-06	9.62E-04
UP_SEQ_FEATURE site:	Interaction with phosphoserine on interacting protein	3	17.65	1.38E-05	YWHAZ, YWHAG, YWHAE	481.84	9.08E-04	9.08E-04	0.014
**Annotation Cluster 4**		**Enrichment Score: 1.47**							
GOTERM_CC_FAT GO: 0048770	pigment granule	3	17.65	0.0047	HSP90AB1, YWHAZ, YWHAE	26.93	0.31	0.046	4.93
GOTERM_CC_FAT GO: 0042470	melanosome	3	17.65	0.0047	HSP90AB1, YWHAZ, YWHAE	26.93	0.31	0.046	4.93
***EC Stress***									
**Annotation Cluster 1**		**Enrichment Score: 2.49**							
GOTERM_BP_FAT GO: 0006091	generation of precursor metabolites and energy	6	3.68	4.03E-07	GOT1, PKM2, ATP5C1, ENO3, SUCLA2, ENO1	27.76	4.31E-05	4.31E-05	4.53E-04
GOTERM_BP_FAT GO: 0006096	glycolysis	3	1.84	5.02E-04	PKM2, ENO3, ENO1	77.07	0.052	0.026	0.56

**Figure 3:**
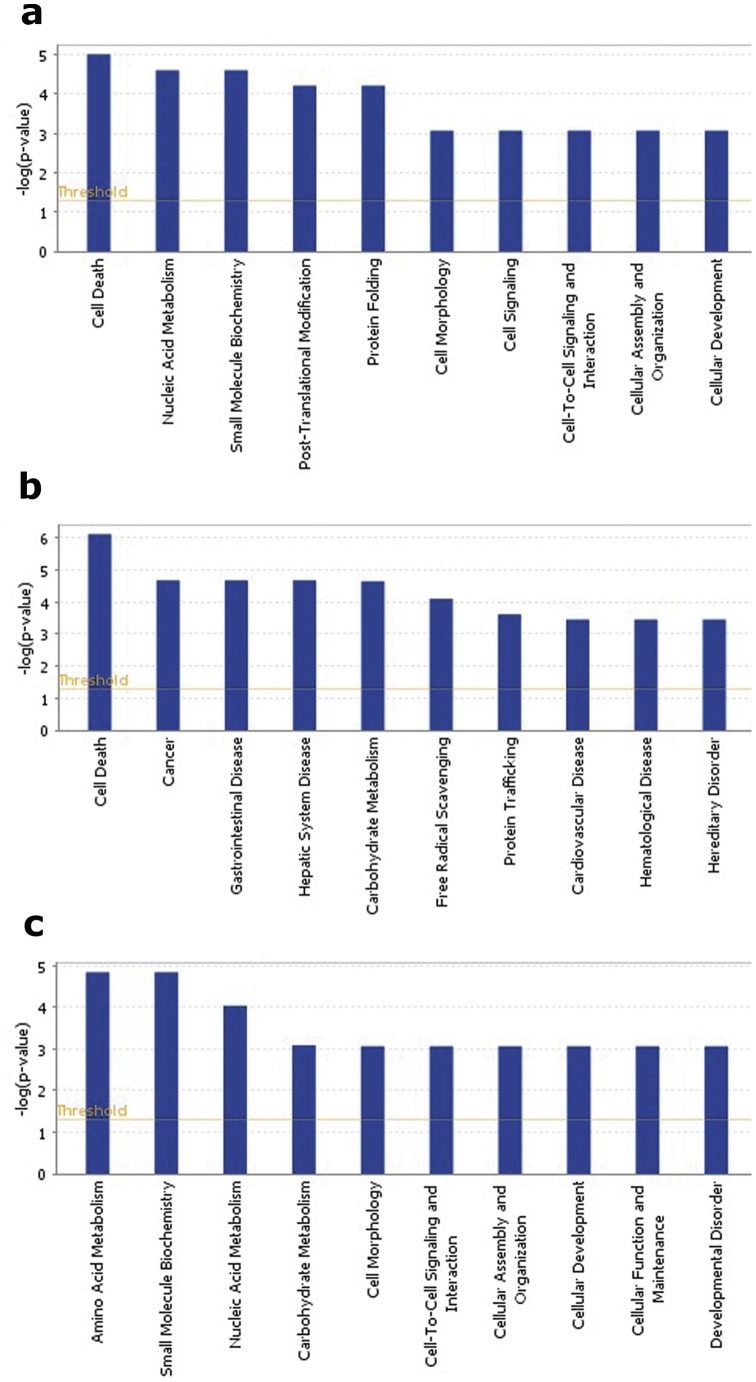
Top ten major biological functionsof regulated proteins comparing EC and IC basal expression (a), IC stress (b), EC stress (c) via IPA analysis. Y-axis represents the –log (P value).

**Figure 4:**
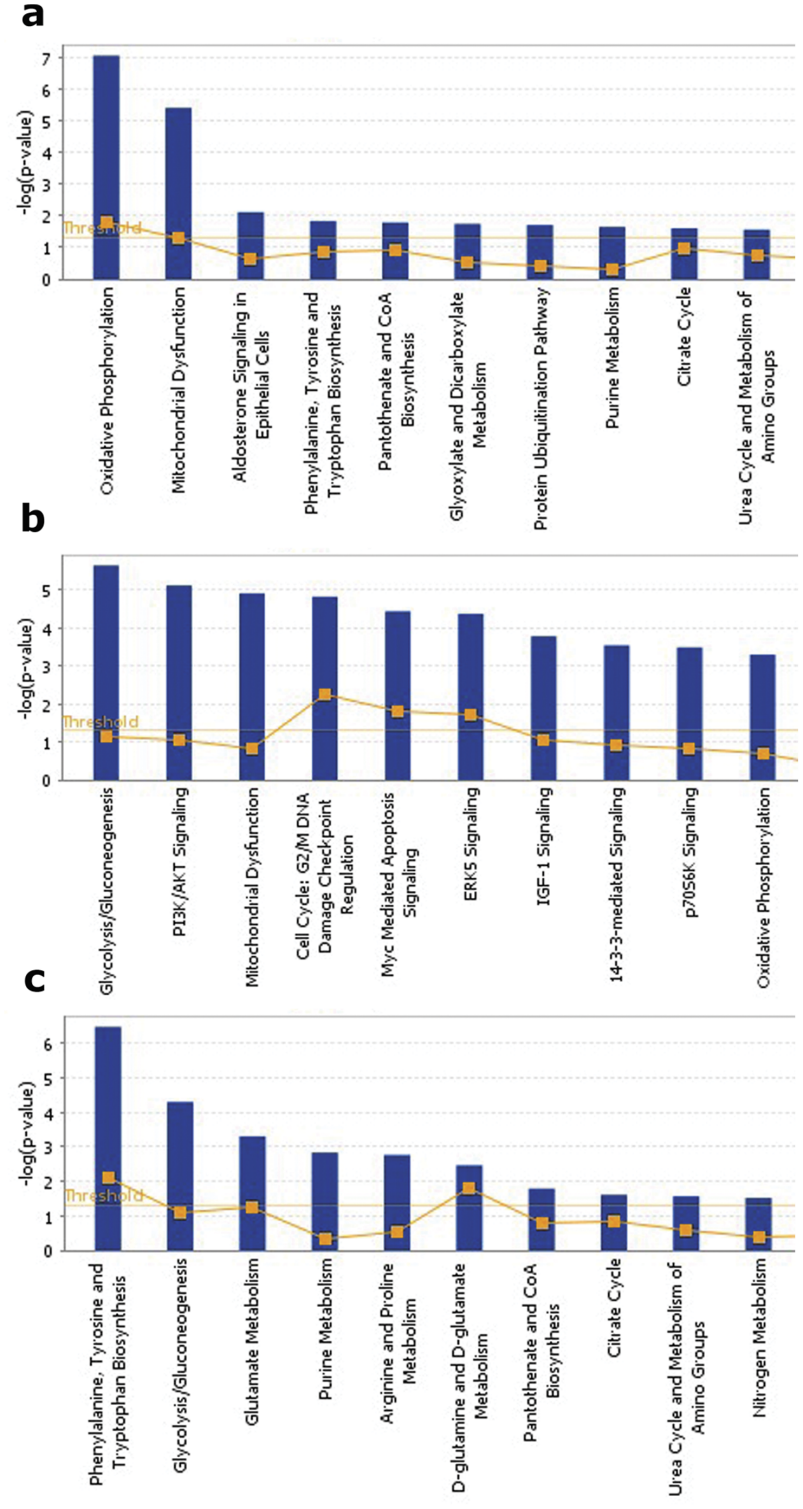
Top ten major canonical pathways of regulated proteins comparing EC and IC basal expression (a), IC stress (b), EC stress (c) via IPA analysis. Y-axis represents the –log (P value).

The IPA analysis yielded top biological functions (with more than one protein) including cell death of neuroblastoma cell line, biosynthesis of purine ribonucleotide, refolding of protein, transport of synaptic vesicles and synthesis of ATP (Figure 3a; Table 4). Note that the protein folding function included heat shock proteins HSPA8 and HSPD1. An additional heat-shock protein (HSP90AB1) was induced by stress in IC rats (see below). Top canonical pathways include oxidative phosphorylation and mitochondrial dysfunction (Figure 4a; Table 5).

**Table 4:** Cellular functions identified *via* IPA analysis.

Category	Function Annotation	P-Value	Molecules	Number of Molecules
***EC/IC Basal Differences***				
Cell Death	cell death of neuroblastoma cell lines	9.76E-06	ATP5A1,ENO1,HSPA8,TUBA1A	4
Nucleic Acid Metabolism	biosynthesis of purine ribonucleotide	2.48E-05	ATP5A1,HSPD1,VDAC1	3
Protein Folding	refolding of protein	6.11E-05	HSPA8,HSPD1	2
Cell Death	cell death of tumor cell lines	8.14E-05	ATP5A1,ENO1,HSPA8,HSPD1,TUBA1A,UQCRFS1,VDAC1	7
Nucleic Acid Metabolism	metabolism of nucleic acid componentor derivative	9.03E-05	ATP5A1,CS,DPYSL2,HSPD1,VDAC1	5
Nucleic Acid Metabolism	synthesis of purine nucleotide	2.09E-04	ATP5A1,HSPD1,VDAC1	3
Cellular Assembly and Organization	transport of synaptic vesicles	8.74E-04	DPYSL2,HSPA8	2
Nucleic Acid Metabolism	synthesis of ATP	9.78E-04	HSPD1,VDAC1	2
***IC Stress***				
Cell Death	cell death of tumor cell lines	7.44E-07	ALB,ATP5A1,ENO1,GAPDH,HSP90AB1,UQCRFS1,VDAC1,YWHAE,YWHAG,YWHAZ	10
Cancer	cholangiocarcinoma	2.08E-05	ALB,HSP90AB1,PGK1	3
Carbohydrate Metabolism	glycolysis of cells	2.21E-05	ENO1,GAPDH,PGK1	3
Free Radical Scavenging	synthesis of reactive oxygen species	7.83E-05	ALB,HSP90AB1,UQCRFS1,VDAC1,YWHAZ	5
Protein Trafficking	targeting of protein	2.39E-04	YWHAE,YWHAG,YWHAZ	3
Cancer	digestive organ tumor	3.03E-04	ALB,DPYSL2,ENO1,HSP90AB1,PGK1,YWHAE,YWHAG,YWHAZ	8
Free Radical Scavenging	production of reactive oxygen species	3.28E-04	HSP90AB1,UQCRFS1,VDAC1,YWHAZ	4
Neurological Disease	Leigh syndrome	3.48E-04	NDUFA9,PDHA1	2
Gene Expression	binding of DNA	3.69E-04	ALB,GAPDH,YWHAE,YWHAG,YWHAZ	5
Nucleic Acid Metabolism	metabolism of nucleic acid component or derivative	3.81E-04	ATP5A1,CS,DPYSL2,PGK1,VDAC1	5
Dermatological Diseases and Conditions	Psoriasis	7.12E-04	CKMT1A,GAPDH,UQCRFS1,VDAC1,YWHAE	5
Inflammatory Disease	acute respiratory distress syndrome	7.26E-04	ALB,GAPDH	2
Cell Death	cell death of neuroblastoma cell lines	8.19E-04	ATP5A1,ENO1,YWHAE	3
***EC Stress***				
Amino Acid Metabolism	catabolism of acidic amino acid	1.42E-05	GLUD1,GOT1	2
Nucleic Acid Metabolism	metabolism of nucleic acid component or derivative	9.03E-05	ATP5C1,DPYSL2,NSF,PKM2,SUCLA2	5
Small Molecule Biochemistry	metabolism of dicarboxylic acid	2.00E-04	GOT1,SUCLA2	2
Nucleic Acid Metabolism	metabolism of nucleoside triphosphate	2.22E-04	ATP5C1,NSF,PKM2	3
Amino Acid Metabolism	synthesis of L-amino acid	3.10E-04	GLUD1,GOT1	2
Carbohydrate Metabolism	glycolysis of cells	8.09E-04	ENO1,PKM2	2

**Table 5:** Canonical pathways identified *via* IPA analysis.

Ingenuity Canonical Pathways	−log(P-value)		Ratio	Molecules		
***EC/IC Basal Differences***						
Oxidative Phosphorylation		7.08	3.14E-02	ATP5A1,UQCRC2,NDUFS2,UQCRFS1,ATP6V1A		
Mitochondrial Dysfunction		5.42	2.30E-02	ATP5A1,UQCRC2,NDUFS2,UQCRFS1		
Aldosterone Signaling in Epithelial Cells		2.12	1.16E-02	HSPA8,HSPD1		
Phenylalanine, Tyrosine and Tryptophan Biosynthesis		1.84	1.49E-02	ENO1		
Pantothenate and CoA Biosynthesis		1.79	1.64E-02	DPYSL2		
Glyoxylate and Dicarboxylate Metabolism		1.75	8.93E-03	CS		
Protein Ubiquitination Pathway		1.71	7.46E-03	HSPA8,HSPD1		
Purine Metabolism		1.65	4.98E-03	ATP5A1,HSPD1		
Citrate Cycle		1.61	1.75E-02	CS		
Urea Cycle and Metabolism of Amino Groups		1.57	1.28E-02	CKMT1A/CKMT1B		
***IC Stress***						
Glycolysis/Gluconeogenesis		5.65	3.08E-02	PGK1,PDHA1,ENO1,GAPDH		
PI3K/AKT Signaling		5.12	2.88E-02	YWHAG,YWHAE,HSP90AB1,YWHAZ		
Mitochondrial Dysfunction		4.91	2.30E-02	PDHA1,NDUFA9,ATP5A1,UQCRFS1		
Cell Cycle: G2/M DNA Damage Checkpoint Regulation		4.82	6.12E-02	YWHAG,YWHAE,YWHAZ		
Myc Mediated Apoptosis Signaling		4.44	4.92E-02	YWHAG,YWHAE,YWHAZ		
ERK5 Signaling		4.37	4.69E-02	YWHAG,YWHAE,YWHAZ		
IGF-1 Signaling		3.78	2.83E-02	YWHAG,YWHAE,YWHAZ		
14-3-3-mediated Signaling		3.54	2.46E-02	YWHAG,YWHAE,YWHAZ		
p70S6K Signaling		3.49	2.26E-02	YWHAG,YWHAE,YWHAZ		
Oxidative Phosphorylation		3.30	1.89E-02	NDUFA9,ATP5A1,UQCRFS1		
***EC stress***						
Phenylalanine, Tyrosine and Tryptophan Biosynthesis		6.48	4.48E-02	ENO1,ENO3,GOT1		
Glycolysis/Gluconeogenesis		4.30	2.31E-02	PKM2,ENO1,ENO3		
Glutamate Metabolism		3.31	2.60E-02	GLUD1,GOT1		
Purine Metabolism		2.83	7.46E-03	PKM2,NSF,ATP5C1		
Arginine and Proline Metabolism		2.77	1.13E-02	GLUD1,GOT1		
D-glutamine and D-glutamate Metabolism		2.47	3.85E-02	GLUD1		
Pantothenate and CoA Biosynthesis		1.79	1.64E-02	DPYSL2		
Citrate Cycle		1.61	1.75E-02	SUCLA2		
Urea Cycle and Metabolism of Amino Groups		1.57	1.28E-02	GLUD1		
Nitrogen Metabolism		1.52	8.40E-03	GLUD1		

**Figure 5:**
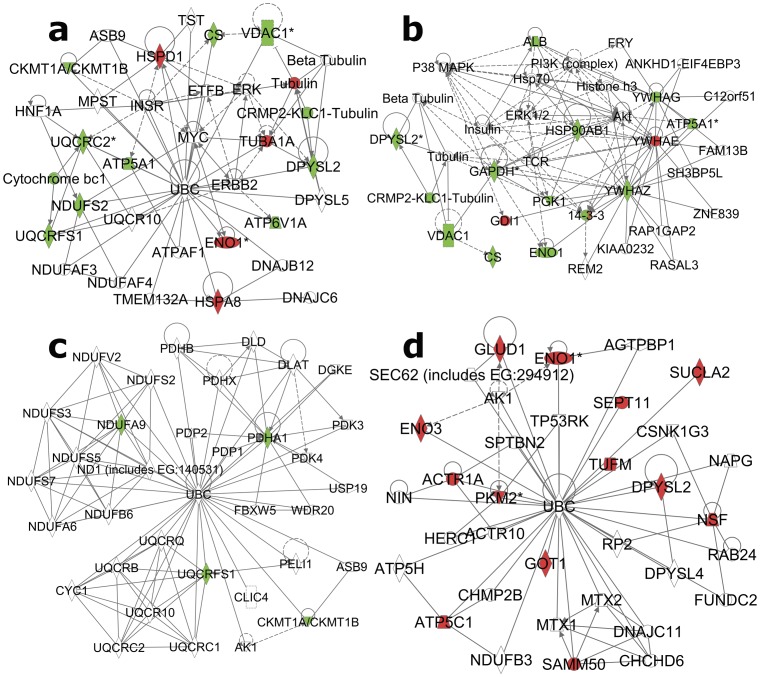
Significant networks identified via IPA analysis of (a) basal EC vs. IC protein expression, (b and c) IC stress, and (d) EC stress. Red symbols represent upregulated and green denote downregulated proteins. Asterisks (*) denote multiple spots mapping to the same protein.

Using the Network Analysis feature, a high scoring network (IPA network score of 40) involving 13 of the 14 identified proteins was detected (Figure 5a). The one feature all 13 proteins shared was binding with ubiquitin. Of these, the two heat-shock proteins HSPA8 and HSPD1, along with ENO1, TUBA1A were upregulated in EC rats. The rest were down-regulated.

### Effect of 30 Min Restraint Stress on Protein Expression in IC Rats

Analysis of the gels for the cytosolic fraction yielded 5 unique proteins from 8 individual differentially regulated spots (Figure 1; Table 1). The membrane fraction yielded 13 proteins from 16 spots (Figure 2; Table 2). One additional spot (YWHAZ) with a P-value of 0.06 was included in the analysis. Proteins identified in two or more discrete spots were ATP5A1 (four spots), GAPDH (four spots) and DPYSL2 (two spots). It is noteworthy that the identified VDAC1 spot was not one of the two VDAC1 spots identified in the analysis of basal EC/IC protein differences as this spot had a lower pI than either of the other two. Of all regulated spots, 23 of 25 were downregulated after stress. A protein involved in glycolysis, ENO1, was lower in IC rats compared to EC rats under basal conditions and decreased further in IC rats subsequent to stress.

When analyzed together, the gene/protein ontology analysis derived 4 clusters of related attributes, namely those relating to glycolysis, mitochondria, 14-3-3 protein family and pigment granule (Table 3). Note that the mitochondrial proteins were lower after stress, bringing them down in the neighborhood of basal EC expression.

The IPA analysis produced top biological functions of death of tumor cell line, cholangiocarcinoma, glycolysis, synthesis of reactive oxygen species, and targeting of protein (Figure 3b; Table 4). A large number of high-scoring canonical pathways were identified, among them being glycolysis, AKT signaling, mitochondrial dysfunction, 14-3-3 signaling, and oxidative phosphorylation (Table 5; Figure 4b). All of the proteins comprising glycolysis and synthesis of reactive oxygen species functions were decreased in IC rats after stress.

The top scoring network (score = 36) drawn from these proteins implicated AKT as a rich and possibly important node relating 13 of the total 19 target proteins together (Figure 5b). These proteins are ALB, ATP5A1, CS, DPYSL2, ENO1, GAPDH, GDI1, HSP90AB1, PGK1, VDAC1, YWHAE, YWHAG, and YWHAZ. Of these, only GDI1 and YWHAE were upregulated with stress. A second lower-scoring network (score = 9) was identified by 4 downregulated proteins (NDUFA9, PDHA1, UQCRFS1, and CKMT1A) that are ubiquitin-interacting proteins (Figure 5c).

### Effect of 30 Min Restraint Stress on Protein Expression in EC Rats

The 2-DE gel analysis for the cytosolic fraction yielded 6 proteins across 9 individual spots (Figure 1; Table 1) and the membrane fraction produced 7 identifiable proteins from 8 spots (one spot was unidentifiable; Figure 2; Table 2). In stark contrast to IC rats, all 16 identifiable spots were upregulated subsequent to stress for EC rats. Of these proteins, PKM2 was identified from 2 separate spots and ENO1 produced a train of 3 regulated spots, including the spot identified in the basal EC/IC and the IC stress analyses. As mentioned above, IC rats had lower basal levels of this protein and sank lower still after stress, while EC rats had higher basal levels and these levels increased even more with stress.

The gene/protein ontology analysis of all 13 spots only derived one cluster of classifications, that cluster being related to glycolysis (Table 3). Although this gene/protein ontology cluster is the same as in IC stressed rats, in this case the glycolysis proteins *increased* in EC rats rather than decreasing as seen with IC rats.

Top unique biological functions were catabolism of acidic amino acids, metabolism of nucleic acid component or derivative, and glycolysis of cells (Figure 3c; Table 4). The IPA analysis top canonical pathways were as follows: phenylalanine, tyrosine and tryptophan biosynthesis, glycolysis, and glutamate metabolism (Figure 4c; Table 5).

As with the basal EC/IC and the IC stress analyses, the network analysis for EC stress identified another network (score = 40) with ubiquitin c as a major node, only in this case, all proteins were upregulated subsequent to stress (Figure 5d). All 13 proteins regulated by stress in EC rats exhibit protein-protein interactions with ubiquitin c.

### Orthogonal Validation of Protein Regulation *Via* Western Blot

Because CBB stains all protein, and because multiple different proteins can migrate to the same position on a 2D gel, it is important to provide orthogonal validation to verify that the 2D gel procedures coupled with mass spectrometry are correctly identifying regulated proteins. Accordingly, three of the most critical regulated proteins were validated *via* 1D Western blot. The antibody specificity of Western blots provides additional confidence in the identification of regulated proteins. Figure S1 shows that in the cases of ENO1 (F(1,10) = 13.9, P<0.005), HSPA8 (F(1,5) = 19.8, P<0.05) and PKM2 (F(1,10) = 12.2, P<0.01), the CBB spot intensity correlated well with antibody-labeled signal on a 1D Western blot.

### ATP Synthase Activity

**Figure 6:**
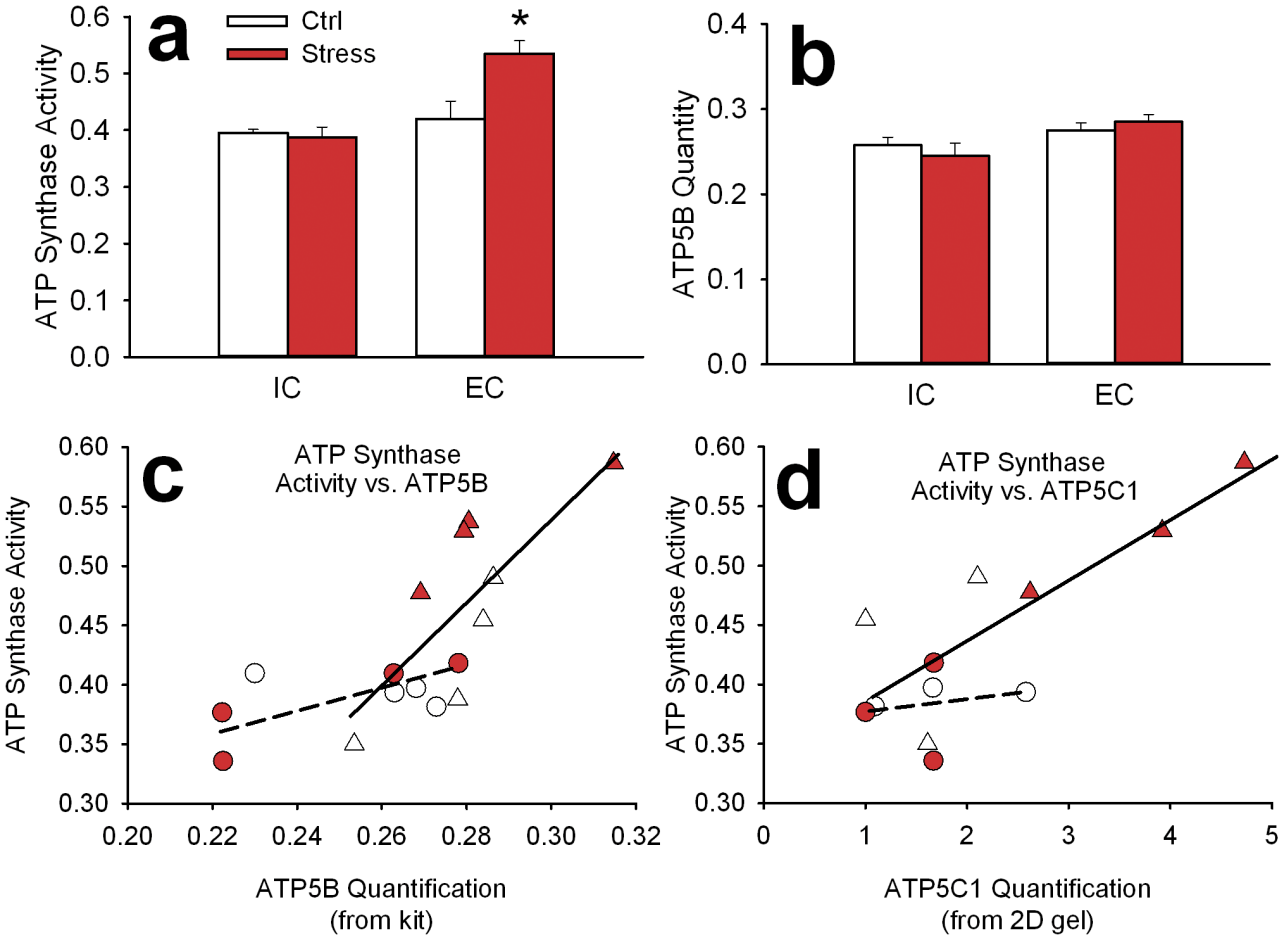
Functional assay of ATP synthase activity and ATP5B quantity. Bars represent mean (± SEM) amount of ATP synthase activity in mM/min (a) and quantity in OD/min (b) of ATP synthase. Asterisk (*) represents statistically significant difference from control. Bottom panels depict correlation of activity and quantity as measured from the kit (c) and activity and quantity of ATP5C1 as measured from the 2D gel (d). Circles represent IC rats and triangles represent EC rats. Controls are white symbols and Stress groups are red. Solid line depicts a significant correlation for EC rats and dashed line depicts a lack of correlation in IC rats.

Validation of differences in protein expression is good practice, but more important than protein expression is protein *function*. Accordingly, because energy producing proteins were most regulated by enrichment and stress, we performed a functional assay of ATP synthase activity. Results of the functional assay demonstrated that EC rat tissue exhibited more ATP synthase activity than IC rats (main effect: F(1,12) = 15.8, P<0.005; Figure 6a) and that, overall, stress increased ATP synthase activity (main effect: F(1,12) = 5.4, P<0.05). Further, there was a significant interaction (F(1,12) = 7.9, P<0.05). The nature of this interaction was such that the Stress main effect increase in activity was driven entirely by EC rats. Regarding quantity (Figure 6b), EC rats had slightly higher levels of ATP synthase (ATP5B) than IC rats (main effect: F(1,12) = 7.0, P<0.05; Figure 6a) but no significant Stress or interaction effect.

Looking more closely at these data (Figure 6c), one can see that there was a significant correlation of ATP synthase activity and ATP5B expression in EC rats (Pearson coefficient = 0.78, P<0.05) but not IC rats (Pearson coefficient = 0.58, n.s.). Similar results were found for ATP synthase activity and ATP5C1 expression in EC (Pearson coefficient = 0.81, P = 0.05; Figure 6d) but not IC rats (Pearson coefficient = 0.19, n.s.). No such relationship was found with expression of the non-CREB target ATP5A1 (data not shown).

### Regulated Energy Metabolism Proteins are CREB Target Genes

**Figure 7:**
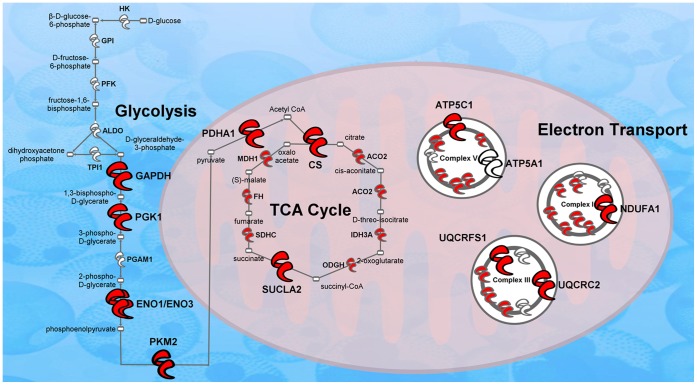
Regulated energy metabolism proteins are CREB targets. The diagram depicts significantly regulated proteins in the glycolysis, TCA and electron transport pathways. The thirteen significantly regulated proteins are shown as large symbols. In addition, 12 of the 13 regulated proteins are from CREB target genes (shown as red symbols).

Less than 30% of promoters for known protein-coding genes are bound by CREB [29]. For the energy metabolism proteins regulated in this study (Figure 7, large symbols), 11 of 13 proteins (84%) are CREB target genes (red symbols). Of the non-energy metabolism proteins identified in this study, only 38% are CREB target genes. This suggests that the low CREB phenotype that mimics the EC protective depression phenotype might be a function of differential energy metabolism.

## Discussion

The current study utilizes the environmental enrichment paradigm, an animal model producing an antidepressant-like behavioral phenotype [11] to investigate differential protein regulation subsequent to an acute psychological stressor in rats. Environmentally enriched rats have been shown previously to be much less responsive with regard to mRNA induction of immediate-early genes 30 min post stress compared to rats raised in isolation [12]. The current experiments further demonstrate robust differences in protein expression patterns between EC and IC rats subsequent to the same stressor. All of the proteins regulated by stress in EC rats were increased in expression while the vast majority of regulated proteins were decreased in expression for IC rats after stress. These results, after only 30 min of stress, highlight the rapid and dynamic nature of protein regulation in response to a changing environment.

### Energy Production

There is increasing evidence from the human literature that energy metabolism plays an important role in major depression [30], [31] and animal studies corroborate these results in the responses to chronic psychological stress [32], [33], [34] and antidepressants [34], [35]. The most striking result of this study was the rapid and robust regulation of proteins involved with glycolysis, the citric acid cycle and mitochondrial respiration. The major product of glycolysis is pyruvate, which is shuttled into the mitochondria to be used in the citric acid cycle which in turn feeds the electron transport chain to produce ATP. Fifteen of the total 35 proteins regulated (42%) in this study were related to energy production. This number also includes creatine kinase, an enzyme that rapidly creates ATP from phosphocreatine storage reservoirs in neurons in response to rapidly increased energy demands [36].

Under basal conditions, EC rats showed lower expression of seven proteins related to energy production and higher expression of only one protein (ENO1). After 30 min of restraint stress, five energy production-related proteins were induced in EC rats, including the ATP synthase protein ATP5C1. A functional ATP synthase assay confirmed that EC rats exhibited an increase in the ability to produce ATP within 30 min of restraint stress. In stark contrast to EC rats, IC rats exhibited a coordinated *decrease* in expression nine energy-related proteins and no increase in the ability to synthesize ATP after stress.

The ENO1 protein is unique among the proteins identified in this study. This protein was identified as 4 discrete spots, each with a high expression level. All had similar molecular weight but different pI. One spot in particular showed higher basal expression in EC rats compared to IC rats and was increased by stress in EC rats yet decreased by stress in IC rats. This opposite modulation of a highly expressed protein draws interest, particularly since this protein has also been identified in proteomic studies of chronic stress [33] and after antidepressant treatment [35].

Creatine kinase is a protein that stores energy as phosphocreatine for a rapid response to increases in demand for ATP [36]. More specifically, this reversible mechanism stores energy when the cell is not being challenged and then quickly releases the stored energy on demand independently from the glycolysis/citric acid/electron transport system. This could be particularly important in light of the opposite modulation of glycolysis and electron transport by stress in EC versus IC rats. In this study, EC rats expressed less mitochondrial creatine kinase under basal conditions, but IC rats exhibited a nearly 3-fold decrease in expression of this protein after 30 min of restraint stress.

### Mitochondria

The citric acid cycle and electron transport functions described above take place in the mitochondria. With such coordinated regulation of these proteins, one must ask the question of whether the ATP synthesis pathways are being regulated individually or if the mitochondria themselves are being regulated. The rapid timecourse of changes in protein levels might make one skeptical, but mitochondrial proteins not directly involved with the citric acid cycle or electron transport are regulated in concert with the energy metabolism proteins. For example, VDAC1 is not directly involved in energy metabolism but is lower in EC basal conditions and is decreased in IC tissue after stress in an identical fashion to the energy metabolism proteins. Similarly, SAMM50, TUFM and GLUD1 are non-energy mitochondrial proteins induced in EC tissue subsequent to stress in a similar fashion to the energy metabolism proteins. Although this idea is speculative at this point, the available evidence here is intriguing.

The idea that major depression in humans might be a function of mitochondrial dysfunction is not novel [37]. There is increasing evidence showing that depressed patients are more likely to have mitochondrial disorders [38], that patients with mitochondrial disorders are more likely to be depressed [39] and that post-mortem brain tissue from patients with major depression exhibit alterations in mitochondrial proteins [31]. It is important to note that these studies focus specifically on genetic perturbations of mitochondrial genomes leading to mitochondrial dysfunction. The current study complements these prior studies by demonstrating that acute psychological stress produces a rapid change in mitochondrial protein expression and that the direction of protein changes are dependent upon the living environment of the subject (EC vs. IC).

### Heat-shock Proteins

Under basal conditions, EC rats had higher expression of the constitutively expressed heat-shock proteins HSPA8 (a.k.a. HSC70 or constitutive heat shock protein 70) and HSPD1 (a.k.a. chaparonin 60). It is interesting to note that the latter is a mitochondrial-associated protein and that a majority of differentially expressed mitochondrial proteins were decreased in EC rats compared to IC rats. However, this protein is also found in the cytosol and the differential expression in this study was found in the cytosolic fraction. In addition to the two constitutive proteins differentially expressed under basal conditions, an additional heat-shock protein, the inducible HSP90AB1 was *decreased* by psychological stress in IC rats. Interestingly, this protein is implicated in signal transduction subsequent to its role in refolding denatured proteins [40], [41]. Another protein likely pulling double duty is ENO1. There is evidence that the *Saccharomyces cervesiae* homolog of the ENO1 protein (HSP48) also functions as a heat shock protein [42].

Although a role for these particular heat-shock proteins in human depression has yet to be identified, preclinical models have identified HSPA8 and HSPD1 as proteins regulated subsequent to *chronic* stress [32], [33]. In addition, HSPD1 is a protein shown to be induced by antidepressant treatment [34]. The fact that the current study found higher expression of both proteins under basal conditions in EC versus IC rats, one might hypothesize that these proteins might underlie the EC diminished response to an acute stressor.

### 14-3-3 Proteins

The 14-3-3 family of proteins has received considerable attention in brain and in relation to neurodegenerative diseases [43]. These proteins can have a multitude of effects on other proteins, particularly those that are phosphorylated at Ser or Thr [44]. Specifically these proteins can play a role in changing the conformation of target proteins, protecting proteins from phosphatases or proteasome degradation, binding of two target proteins, and targeting/sequestering proteins in specific cellular compartments [45]. With such high expression and varied functions, these proteins are very important to the functioning of the cell. Indeed, a recent paper identified YWHAZ as a “switchboard” gene for neuropsychiatric disorders [30]. Thus, although the functional consequences have yet to be elucidated, the dynamic regulation of YWHAG, YWHAE and YWHAZ after stress in IC rats is intriguing.

### DPYSL2 (CRMP2)

The Allen Brain Atlas shows that the mRNA for DPYSL2 exhibits targeted expression specifically to the NAcc compared to that of the dorsal striatum in mouse brain [46]. The protein DPYSL2 was found to be differentially regulated in EC and IC rats, with EC rats showing *increased* expression after psychological stress and IC rats showing *decreased* expression. Although the dominant function of this protein in adult brain has not been conclusively identified, it is clear that this protein is somehow involved with Alzheimer’s disease [47], [48] and possibly schizophrenia [31]. Relating to depression, this protein has appeared in preclinical proteomic studies of chronic stress and also antidepressant treatment [32], [33], [34].

The protein itself plays an important role in axon guidance and neurite outgrowth in developing neurons, as well as vesicle trafficking functions [48], [49]. Interestingly, this protein has also been shown to affect current density of N-type voltage-gated Ca^2+^ channels, presumably by controlling localization [50]. This effect has been shown to be functional, as evidenced by a decrease in vesicular neurotransmitter release [51]. Further, this protein has also been shown to regulate trafficking of NMDA receptors, providing an attractive target for preventing excitotoxicity [52].

### Ubiquitin c

The utility of IPA’s Network analysis is to identify proteins and systems that may not be directly identified as being regulated but still play an important role in regulating other proteins. Three of the four identified networks (Figure 5) were centered on ubiquitin protein-protein interactions (26 of the total 35 proteins). Ubiquitin conjugation has been shown to be involved with proteasomal degradation, but an estimated 50% of ubiquitin sites are not proteasomal and presumably represent signaling modifications [53]. It is interesting that all 4 ubiquitin targets in the IC Stress network (Figure 5c) were downregulated by stress and all 13 ubiquitin target proteins in the EC Stress network were upregulated. Although ubiquitin c itself is too small to be detected on these gels, these networks suggest that EC and IC rats may show differential ubiquitin regulation. A recent bioinformatics paper has identified ubiquitin c as a possible “switchboard” gene for psychiatric disorders including depression [30].

### Other Findings

Interestingly, one recent investigation of protein-protein interactions in human schizophrenic, bipolar and major depression prefrontal cortex tissue identified a very tight network of 12 proteins [30]. Of the 12 proteins identified by Lee et al., 6 were identified in the current study (HSPA8, HSPD1, HSP90AB1, GAPDH, TUBA1A and YWHAZ). Another protein in the network, ubiquitin c, is a protein too small to be seen on the 2DE gels used here but this protein was identified as a hub in multiple networks of the current study. For the UBC-centric network of EC/IC basal differences in protein expression (Figure 5a), EC rats had higher expression of only 4 of the 13 proteins. Of these 4 proteins, 3 were members of the network identified by Lee et al. (HSPA8, HSPD1 and TUBA1A).

Three of the networks identified in Figure 5 (a, b and d) were very high-scoring networks (scored 36 or better), each comprising a clear majority of regulated proteins in each analysis. These extensively-regulated networks suggest coordinated regulation of protein degradation (see ubiquitin discussion above) or kinase signaling cascades. The kinases themselves are typically regulated by phosphorylation, a modification not easily discerned when looking at total protein expression. As a result, the kinases themselves are not revealed, but network analyses can point to likely kinase involvement. Figure 5a shows ERK as a hub likely to be involved in EC/IC basal differences. Prior research provides ample evidence that ERK plays a role in depression [54], [55]. Figure 5b also identifies ERK as a likely player in IC control vs. stress along with other known key signaling cascades P38 MAPK and PI3K/AKT. These results are also supported by literature demonstrating the importance of these cascades in depression [56], [57]. Additionally, histone H3 was identified as a possible player.

### Role for CREB

Our prior research has shown a clear role for CREB activity in producing the protective depression and addiction phenotypes seen with environmental enrichment [11], [12], [13], [14], [15], [16], [58]. Specifically, EC rats exhibit a very unique behavioral phenotype: they show less spontaneous locomotor activity than IC rats, show enhanced responsiveness to cocaine- and amphetamine-stimulated locomotor activity and conditioned place preference [11], [59], [60], exhibit an antidepressant-like phenotype in sucrose preference, social interaction and forced swim test paradigms [11], yet EC rats exhibit an anxio*genic*-like phenotype in sucrose neophobia and cold-stress-induced defecation paradigms [11]. This very specific phenotype can be recapitulated by viral-mediated overexpression of a dominant-negative mutant CREB [13], [16], [18], an endogenous dominant-negative inhibitor of CREB transcription (*i.e.* ICER) [13], or by knocking down CREB expression with an shRNA vector [11] in the NAcc. Additionally, we showed that EC rats have less basal pCREB (*i.e.* active CREB) in the NAcc [11], an effect replicated by the Zhu laboratory [61].

Because a clear majority of nuclear-encoded mitochondrial genes are CREB target genes [29], and we know that CREB is an important mediator of the enrichment phenotype [11], it is not surprising that approximately half of the differentially-expressed proteins from EC and IC NAcc are mitochondrial proteins. When examining glycolysis proteins with TCA cycle proteins and electron transport proteins, 11 of the 13 regulated proteins are CREB target genes. Although further investigation is necessary to determine if these are the specific CREB target genes mediating the EC phenotype, the current data are consistent with the CREB hypothesis of environmental enrichment [11] and point to energy metabolism as a possible proximal mechanism of this phenotype.

## Conclusions

Although other studies have examined the effects of chronic stress in other brain regions [32], [33], [34], this is the first such study to assess rapid and dynamic changes in protein levels after acute stress in the NAcc in a rat model of resistance to depression-like behavior. Our results identify rapid and opposite changes in proteins of EC and IC rats. The most robust findings are proteins related to energy metabolism (e.g. glycolysis, citric acid cycle and electron transport). Concomitant changes in other mitochondrial proteins not directly related to these functions suggest that the regulation might be at the mitochondrial level rather than individual proteins. These results further support the mitochondrial hypothesis of major depression [37]. In addition, the differences in energy metabolism, along with changes other proteins, provide possible new avenues for pharmacotherapeutic strategies for the treatment of stress and/or depression.

## Supporting Information

Figure S1 Orthogonal validation of expression changes *via* correlation of 2D gel CBB normalized intensity and 1D antibody-labeled Western blot normalized intensity for (a) ENO1 (R^2^ = 0.58), (b) HSPA8 (R^2^ = 0.83) and PKM2 (R^2^ = 0.55).
(TIF)
