# Supplementary figures and images for: Dynamic Proteomics of Nucleus Accumbens in Response to Acute Psychological Stress in Environmentally Enriched and Isolated Rats
Source: PLoS One. 2013 Sep 9;8(9):e73689. doi: 10.1371/journal.pone.0073689 (PMC3767735; doi:10.1371/journal.pone.0073689)

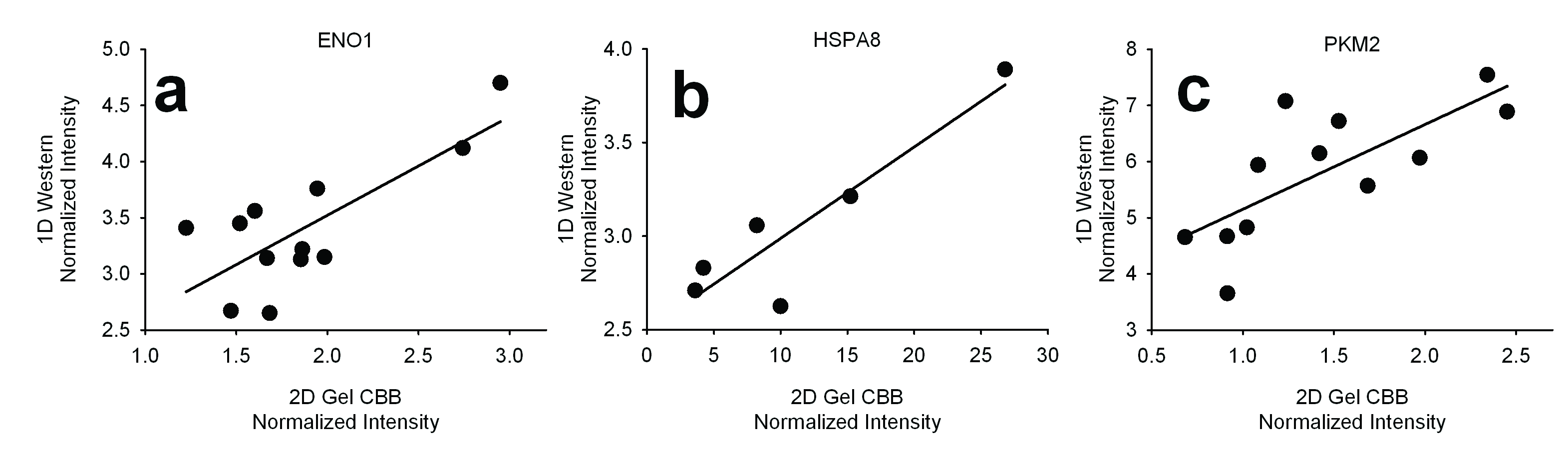

Supplement: Figure S1 — Orthogonal validation of expression changes via correlation of 2D gel CBB normalized intensity and 1D antibody-labeled Western blot normalized intensity for (a) ENO1 (R2 = 0.58), (b) HSPA8 (R2 = 0.83) and PKM2 (R2 = 0.55). (TIF) [file pone.0073689.s001.tif]
